# The mitochondrial BCKD complex interacts with hepatic apolipoprotein E in cultured cells in vitro and mouse livers in vivo

**DOI:** 10.1007/s00018-023-04706-x

**Published:** 2023-02-07

**Authors:** Johanna Rueter, Gerald Rimbach, Christian Treitz, Anke Schloesser, Kai Lüersen, Andreas Tholey, Patricia Huebbe

**Affiliations:** 1 Institute of Human Nutrition and Food Science, University of Kiel, Hermann-Rodewald-Strasse 6, 24118 Kiel, Germany; 2 Institute of Experimental Medicine, University of Kiel, Niemannsweg 11, 24105 Kiel, Germany

**Keywords:** Protein–protein interaction, Dietary restriction, Voltage-dependent anion-selective channel 1, Branched-chain amino acids, Mitochondrial function, Interactome

## Abstract

**Background and aims:**

Apolipoprotein E (APOE) is known for its role in lipid metabolism and its association with age-related disease pathology. The aim of the present work was to identify previously unknown functions of APOE based on the detection of novel APOE protein–protein interaction candidates.

**Approach and results:**

APOE targeted replacement mice and transfected cultured hepatocytes expressing the human isoforms APOE3 and APOE4 were used. For 7 months, APOE3 and APOE4 mice were fed a high-fat and high-sugar diet to induce obesity, while a subgroup was subjected to 30% dietary restriction. Proteomic analysis of coimmunoprecipitation products from APOE mouse liver extracts revealed 28 APOE-interacting candidate proteins, including branched-chain alpha-keto acid dehydrogenase (BCKD) complex subunit alpha (BCKDHA) and voltage-dependent anion-selective channel 1 (VDAC1). The binding of APOE and BCKDHA was verified in situ by proximity ligation assay in cultured cells. The activity of the BCKD enzyme complex was significantly higher in obese APOE4 mice than in APOE3 mice, while the plasma levels of branched-chain amino acids and mTOR signalling proteins were not different. However, the protein–protein interaction with VDAC1 was strongly induced in APOE3 and APOE4 mice upon dietary restriction, suggesting a prominent role of APOE in mitochondrial function.

**Conclusions:**

The protein–protein interactions of APOE with BCKDHA and VDAC1 appear to be of physiological relevance and are modulated upon dietary restriction. Because these are mitochondrial proteins, it may be suggested that APOE is involved in mitochondria-related processes and adaptation to hepatic energy demands.

**Supplementary Information:**

The online version contains supplementary material available at 10.1007/s00018-023-04706-x.

## Introduction

Apolipoprotein E (APOE) is polymorphic in humans, and the three alleles (*ε*2, *ε*3, and *ε*4) encode three isoproteins (APOE2, APOE3, and APOE4) that differ by one or two amino acids. APOE is mainly synthesized in the liver, but most research is being performed on the role of APOE in the brain, since APOE4 is an independent risk factor for Alzheimer’s disease and age-related mortality [1, 2]. However, it has been suggested that the *APOE* gene can have pleiotropic effects on health and survival [3] and that the *ε4* allele may be beneficial under certain conditions, such as vitamin D inadequacy [4], but detrimental in elderly individuals.

Beyond its role in lipid metabolism, APOE was previously associated with immune function [5–7], antiviral defence [8], the regulation of gene transcription [9, 10], and mitochondrial function. Numerous studies have emphasized the influence of APOE isoforms on mitochondria in the brain, including their influence on changes in ATP levels [11–13], mitochondrial respiration, the expression of protein complexes of the oxidative phosphorylation system [14, 15], and mitochondrial fusion and fission proteins [12, 14, 16].

The diversity of the biological processes in which APOE may be involved indicates that there are other yet-unknown functions that remain to be discovered. One approach to identifying potential novel functions of a protein is untargeted detection of protein–protein interactions. By identifying functionally characterized interacting proteins, insights into new metabolic pathways in which the bait protein is involved can be gained. Therefore, the aim of this work was to identify proteins that interact with APOE and to characterize the physiological significance of these interactions to deduce noncanonical functions of APOE beyond lipid metabolism.

## Methods

### Cell culture

#### Culture of human hepatoma cells

Huh7 and HepG2 cells (both human hepatoma cell lines; Institute of Applied Cell Culture, Munich, Germany) were cultured in RPMI 1640 medium (4.5 g/l glucose, 2 mM l-glutamine, 1 mM sodium pyruvate, 10 mM HEPES, and 1.5 g/l NaHCO_3_) and DMEM (4.5 g/l glucose, 3.9 mM l-glutamine, 1 mM sodium pyruvate, and 3.7 g/l NaHCO_3_), respectively. Cell culture media were supplemented with 10% foetal bovine serum (FBS; Gibco, Thermo Fisher Scientific, Waltham, USA) and 1% penicillin/streptomycin. Cells were grown in 5% CO_2_ at 37 °C in a humidified atmosphere. Except for FBS, all cell culture reagents were purchased from PAN Biotech (Aidenbach, Germany).

#### APOE3 and APOE4 plasmid DNA construction, transformation, and transient transfection of human hepatoma cells

Plasmids containing human *APOE3* and *APOE4* DNA were used to study genotype-specific effects of the APOE protein in cultured cells. The human *APOE3* (NM_000041)-expressing pCMV6-AC vector was purchased from OriGene (SC319433, Rockville, USA). The *APOE4* vector was constructed by custom mutagenesis of SC319433 by a T-to-C substitution at nucleotide position 388, resulting in an amino acid alteration of cysteine to arginine in codon 130, corresponding to codon 112 in the mature APOE protein. Transformation, plasmid DNA isolation, and transient transfection were performed as described in Dose et al. [17]. The cells were harvested and processed 48 h after transfection for the following analyses, except for immunostaining experiments, in which cells were harvested 24 h after transfection.

#### BT2 treatment

HepG2 cells were treated with 3,6-dichloro-benzo[b]thiophene-2-carboxylic acid (BT2; Biomol, Hamburg, Germany) to increase endogenous BCKD activity. Twenty-four hours after transfection, the cells were treated with 0.25 mM BT2 in 1 g/l glucose DMEM containing 2% FBS for 24 h.

### APOE targeted replacement (TR) mice

To study the influence of the APOE isoforms in vivo, APOE TR mice were used. APOE TR mice were developed in the laboratory of Nobuya Maeda at the University of North Carolina as described in Sullivan et al. [18] and are homozygous for human *APOE3* or *APOE4* on a C57BL/6NTac background (strains: B6.129P2-*Apoe*^tm2(APOE*3)Mae^ N8 and B6.129P2-*Apoe*^tm3(APOE*4)Mae^ N8, respectively). APOE TR mice are a well-established model for studying human *APOE* genotypes, as has been shown in several studies [4, 11, 19].

The mouse study was approved by the local animal ethics committee and was performed in compliance with German animal welfare regulations. Thirty-two APOE TR mice were purchased from Taconic (Ry, Denmark) at the age of 6–7 weeks, and eight female and 24 male APOE3 and APOE4 mice were used. The female mice were housed in groups of four, while the male mice were housed individually to reduce social stress due to rivalry. The mice were housed under constant environmental conditions on a 12-h light/dark cycle at 22 °C in 55–60% humidity. After a two-week adaptation period, the mice were fed a semisynthetic diet rich in fat and sugar (HFSD; TD88137 modified, Ssniff, Soest, Germany). All mice had free access to water, and the female mice also had unlimited access to food. The male mice were divided into two groups per genotype: one group had ad libitum (AL) access to food, whereas the other was subjected to dietary restriction (DR) and consumed only 70% of the diet consumed by the AL group. DR was gradually introduced by reducing the amount of feed by 5% per week after the adaptation phase until the amount provided was 70% of that provided the AL-fed mice. One AL-fed APOE4 mouse dropped out and had to be euthanized in compliance with animal welfare regulations. The respiratory exchange ratio (RER) was measured at four time points as described previously [20]. At 9–10 months of age, the mice were killed and blood, liver, and muscle were removed. Plasma and tissue were snap-frozen in liquid nitrogen and stored at − 80 °C until analysis.

### Preparation of tissue lysates for coimmunoprecipitation and Western blotting

Whole-cell lysates were prepared by lysing cells and tissues in NP40 lysis buffer supplemented with protease and phosphatase inhibitors. (The compositions of all buffers are listed in Table S1). Mouse tissue was homogenized in a TissueLyser II (Qiagen, Hilden, Germany) for 4 min at 25 Hz, and the lysates were incubated on ice for 30 min and centrifuged for 20 min at 4 °C and 12,000×g. Supernatants (total protein) were stored at − 80 °C until further analysis, and protein concentrations were determined with a Pierce BCA Protein Assay Kit (Thermo Fisher Scientific, Waltham, USA).

### Coimmunoprecipitation

APOE coimmunoprecipitation (co-IP) was performed on liver tissue lysates from APOE TR mice followed by Western blotting and liquid chromatography–mass spectrometry (LC–MS) to analyse APOE protein–protein interactions and to detect yet-unknown interacting proteins. APOE was precipitated from mouse liver tissue lysates or cell lysates using an antibody against APOE. In detail, 500–1000 µg of protein from liver homogenates of APOE TR mice or transfected Huh7 cells was used. The samples were precleared with 20 µl of washed Protein G Sepharose beads (GE Healthcare, Uppsala, Sweden) for 30 min to remove nonspecific proteins bound to the beads. Supernatants were incubated with 5 µg of a monoclonal anti-APOE antibody (sc-13521, Santa Cruz, Dallas, USA) overnight, and a negative control sample without antibody (no AB) was also established. The next day, the protein–antibody complexes were incubated with 50 µl of washed beads. After 60 min, the complexes were centrifuged for 1 min at 12,000×*g*, and the supernatant was collected as the post-IP supernatant. The beads were then washed with NP40 lysis buffer several times. All incubation steps were carried out at 4 °C on a rotating wheel at 20 rpm. The method of elution was dependent on the subsequent experiment, and the detailed methods of the following LC–MS and Western blot analyses are described in Supplementary Material and Methods.

To identify potential binding partners of APOE, co-IP proteomic analyses were performed in two female APOE TR mice (1 × APOE3 and 1 × APOE4) with two different APOE antibodies (sc-13521 and sc-390925, Santa Cruz, Dallas, USA), resulting in a total of four APOE co-IPs performed and analysed by MS. The remaining six female mice were only used for method validation (co-IP MS). To investigate the influence of the APOE isoform and the effect of diet on identified binding partners, co-IP Western blot experiments were performed in the male APOE TR mice (five–six animals per APOE isoform and diet).

### Western blotting

Analysis of protein expression in whole-cell lysates and of APOE and interacting proteins in co-IP samples was performed by Western blotting. Western blot analyses were performed, and images were analysed as previously described [20]. Equal amounts of protein or equal volumes of co-IP samples were loaded onto gels. The suppliers of the primary and secondary antibodies are listed in Table S2.

### BCKD enzyme activity assay

The activity of the BCKD enzyme complex was determined according to the protocol of Nakai et al. [21] with modifications. Both the actual activity (inhibition of BCKD kinase activity) and the total activity (complete dephosphorylation) were determined and used to calculate the percent activity, which reflects the active BCKD complex. The compositions of the buffers were partially modified and are listed in Table S1. The liver tissue extract was prepared as described in Nakai et al. [21], and the protein concentration was determined using the BCA assay. To determine the total activity of the BCKD complex, an aliquot of the tissue extract was incubated with lambda protein phosphatase (final concentration 2000 units/ml; sc-200312, Santa Cruz, Dallas, USA) and manganese chloride (final concentration 2 mM) for 20 min at 37 °C. After incubation, insoluble material was removed by centrifugation at 12,000×*g* for 3 min. The total volume of each reaction was 600 µl and consisted of 300 µl of 2 × assay buffer, 108 µl of double-distilled water, and 180 µl of tissue extract (with or without lambda phosphatase treatment). The mixture was prewarmed to 30 °C for 10 min and then transferred to a cuvette and placed in a spectrophotometer (UV/Vis Spectrophotometer DU800, Beckman Coulter, Krefeld, Germany). The absorbance of the reaction was measured at 340 nm (with background correction at 500 nm) for 10 min to determine the baseline activity. Subsequently, 12 µl of prewarmed α-ketoisovalerate solution (final concentration 1 mM) was added and mixed thoroughly to start the reaction. The absorbance was measured for 20 min at 340 nm (with background correction at 500 nm). Enzyme activity was calculated using the following equation: *c* [mol/l/min] = ((∆ Abs)/(ε*d)), where ∆ Abs is the change in absorbance per min; ε molar absorptivity of product (NADH: 6.22 × 10^3^ l/(mol cm)); and d path length of light through sample (1 cm). One unit of BCKD complex catalyses the conversion of 1 µmol of NADH per minute. The calculated enzyme activity was related to the protein concentrations in the tissue extracts so that the total BCKD complex activity was expressed in nmol/min/g protein.

### Quantification of plasma amino acid concentrations

Quantification of amino acid concentrations in plasma samples from male APOE TR mice was performed by HPLC. Plasma samples were prepared according to Gürke et al. [22] by mixing 20 µl of plasma with 5 µl of 5% sulfosalicylic acid and 5 µl of 6 mM norvaline, which served as an internal standard. After incubation on ice for 30 min, the mixture was centrifuged at 16,000×*g* for 10 min at 4 °C, and the supernatant was diluted 1:5 with ice-cold borate buffer. Derivatization was performed as described by Zhang et al. [23] by mixing 30 µl of the diluted sample with 50 µl of OPA derivatization reagent (prepared from 5 mg of OPA in 500 µl of ddH_2_O, 1 ml of borate buffer, and 27 µl of ethanethiol). Standard amino acid solutions were employed to determine the retention times of the individual amino acids. Calibration curves were generated using a concentration range (2.5–50 µM) of the standard amino acids. Amino acids were analysed by HPLC according to the method described by Schuster et al. [24] with modifications using an Agilent 1100 HPLC system equipped with a diode array detector (338 nm) and a more sensitive fluorescence detector (Ex: 337 nm, Em: 454 nm). Separation was performed on an Eclipse XDB-C18 column (4.6 × 250 mm; 5 µm; all equipment from Agilent Technologies, Les Ulis, France) with a gradient of 60 mM sodium acetate and 0.6% tetrahydrofuran (mobile phase A) and acetonitrile, 0.1 M sodium acetate, and methanol (14:4:1; v/v/v; mobile phase B). The gradient parameters are listed in Table S3. The oven temperature was set to 42 °C, and the injection volume was 10 µl.

### Measurement of hepatic ATP levels

Approximately 10 mg of frozen liver tissue was homogenized in 150 µl of ATP lysis buffer (Table S1) in a precooled Dounce homogenizer (P7734, Sigma, Steinheim, Germany). The homogenate was centrifuged at 13,000×*g* for 2 min, the supernatant was transferred into a new tube, and the protein concentration was determined with the BCA assay. The supernatant was mixed immediately with perchloric acid (final concentration 1 M) and incubated on ice for 5 min to inactivate ATPases. The homogenate was centrifuged at 13,000×*g* for 2 min, the supernatant was transferred to a new tube, and 2 M KOH was added to neutralize the homogenate (final pH 6.5–8). The neutralized homogenate was centrifuged at 13,000×*g* for 15 min, and the supernatant was collected and stored at − 80 °C until ATP measurement. All centrifugation steps were carried out at 4 °C. ATP was quantified by bioluminescence using an ATP determination kit (A22066, Thermo Fisher Scientific, Waltham, USA). ATP measurement was taken following the manufacturer’s instructions by measuring the bioluminescence in a Tecan infinite F200 microplate reader (Tecan, Groedig, Austria). The ATP content was related to mg protein and expressed in nmol ATP/mg protein.

### Proximity ligation assay

Proximity ligation assay (PLA) was conducted in situ in intact fixed Huh7 cells to detect protein–protein interactions. In brief, Huh7 cells were seeded, transfected to express APOE3 or APOE4, stained with 200 nM MitoTracker Red CMXRos (Cell Signaling, MA, USA), fixed, and permeabilized, and PLA was then performed according to the manufacturer’s protocol. A detailed description of the method, including modifications, is given in Supplementary Material and Methods.

### Statistical analysis

Statistical calculations were done with GraphPad Prism 9.1.0 (GraphPad Software, USA). Data were analysed for normality of distribution with the Shapiro‒Wilk test, and homogeneity of variance was tested with the Brown–Forsythe test. For comparisons between two groups, unpaired t test was conducted (parametric data). One-way ANOVA followed by Šídák’s or Tukey’s post hoc multiple comparison test was used for multiple pairwise comparisons. Repeated measures ANOVA was applied for comparison of body weight gain. For data with heterogeneity of variance, Welch’s ANOVA followed by Dunnett’s T3 multiple comparison test was performed. For nonnormally distributed data, the Kruskal‒Wallis test followed by Dunn’s multiple comparison test was performed. Two-way ANOVA was performed to evaluate BCKD complex enzyme activity and ATP measurement data, and the effects of diet (AL and DR), isoform (APOE3 and APOE4) and the diet x isoform interaction were analysed.

All results are shown as the means ± SEMs. Significance was accepted at *p* < 0.05 and is indicated with an asterisk (*) in the figures.

## Results

### APOE interacts with mitochondrial proteins

#### APOE coimmunoprecipitation studies identified a large number of proteins potentially associated with APOE

APOE co-IP followed by LC–MS was performed on liver tissue lysates from two female APOE TR mice. Twenty-eight potential APOE-interacting proteins were selected as candidates for further investigation from a set of more than 300 proteins (complete list, Table S5; 28 candidates, Table S6). The selection was based, among other parameters, on the enrichment of the potential binding partners in the co-IP eluate compared with the noAB IP control and is described in Supplementary Material and Methods. Interestingly, a high number of mitochondrial proteins showed a strong interaction with APOE, although APOE is known as a classical secretory protein.

#### APOE protein–protein interactions in Huh7 cells

**Fig. 1:**
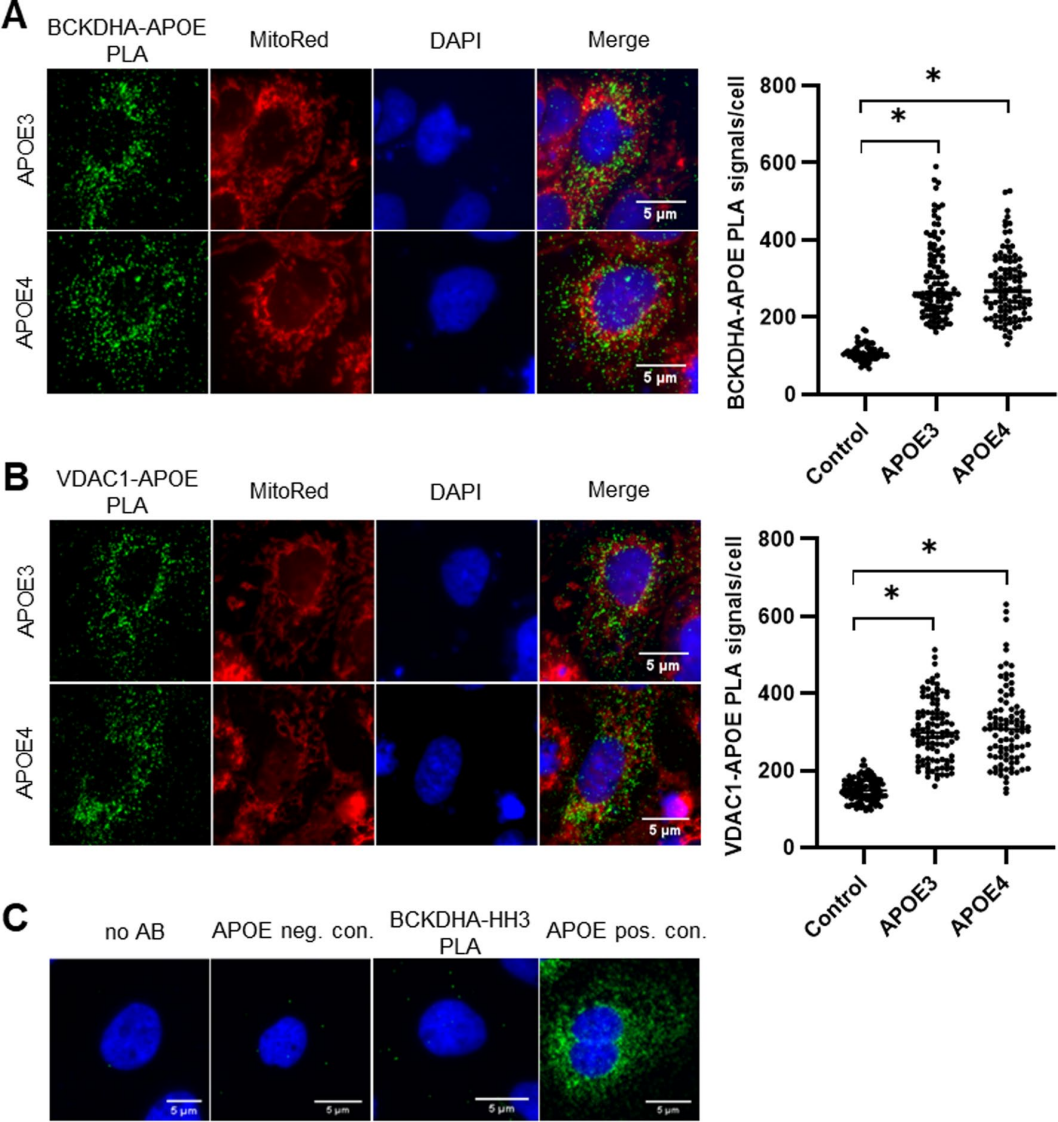
APOE interacts with BCKDHA and VDAC1 in situ in APOE3- and APOE4-transfected Huh7 cells. Representative images of PLAs performed in Huh7 cells. The green dots represent PLA signals, nuclei are stained blue with DAPI, and mitochondria are stained red. Magnification × 400; scale bar, 5 µm. The interaction of APOE with BCKDHA (**A**) and VDAC1 (**B**) was confirmed in APOE3- and APOE4-transfected Huh7 cells. The number of PLA signals per cell did not differ between the APOE3 and APOE4 groups for either the BCKDHA–APOE or the VDAC1–APOE (both *p* > 0.999) interaction. The PLA signals per cell in the control group (= nontransfected cells) were significantly fewer than those in the APOE3 and APOE4 groups for both interactions (all *p* < 0.001; Kruskal‒Wallis test, Dunn’s multiple comparison test). *(*p* < 0.05) indicates a significant difference between samples. (**C**) Different controls were included to avoid misinterpretation of nonspecific binding

Two of the 28 candidates were independently confirmed by co-IP followed by Western blotting and PLA: branched-chain alpha-keto acid dehydrogenase subunit alpha (BCKDHA) and voltage-dependent anion-selective channel 1 (VDAC1) (Fig. S1 and Fig. 1). BCKDHA is a subunit of the BCKD enzyme complex, which is involved in the degradation of branched-chain amino acids (BCAAs) in the mitochondrial matrix. VDAC1 is localized in the outer mitochondrial membrane (OMM) and is responsible for the exchange of molecules between the mitochondrial matrix and the cytosol or other organelles. Representative PLA images of the association of APOE with BCKDHA and VDAC1 in Huh7 cells transfected with the APOE3 or APOE4 expression vector are shown in Fig. 1A, B. APOE3- and APOE4-transfected hepatocytes showed similar numbers of BCKDHA–APOE PLA signals per cell. Likewise, no difference in the number of PLA signals per cell was detected for the VDAC1–APOE interaction, in line with the results of co-IP/Western blotting in cultured hepatocytes (Fig. S1). Overexpression of APOE increased the number of PLA signals per cell by two- and threefold for the BCKDHA–APOE and VDAC1–APOE interactions, respectively. All negative control groups (no AB, APOE negative control, and BCKDHA–HH3 PLA; Fig. 1C) showed a lower number of PLA signals per cell than the test PLA experiments, and a high number of PLA signals were observed in the APOE single recognition control (APOE positive control) group.

### Biological relevance of APOE protein–protein interactions in vivo in the livers of APOE TR mice

The biological relevance of protein interactions was then investigated in 23 male APOE TR mice subjected to diet-induced obesity and DR. AL-fed mice significantly gained body weight, and this gain was more pronounced in the presence of APOE3 than in the presence of APOE4, whereas DR-fed mice maintained a lean phenotype (Fig. S2).

#### APOE interacts in vivo with BCKDHA and VDAC1

**Fig. 2:**
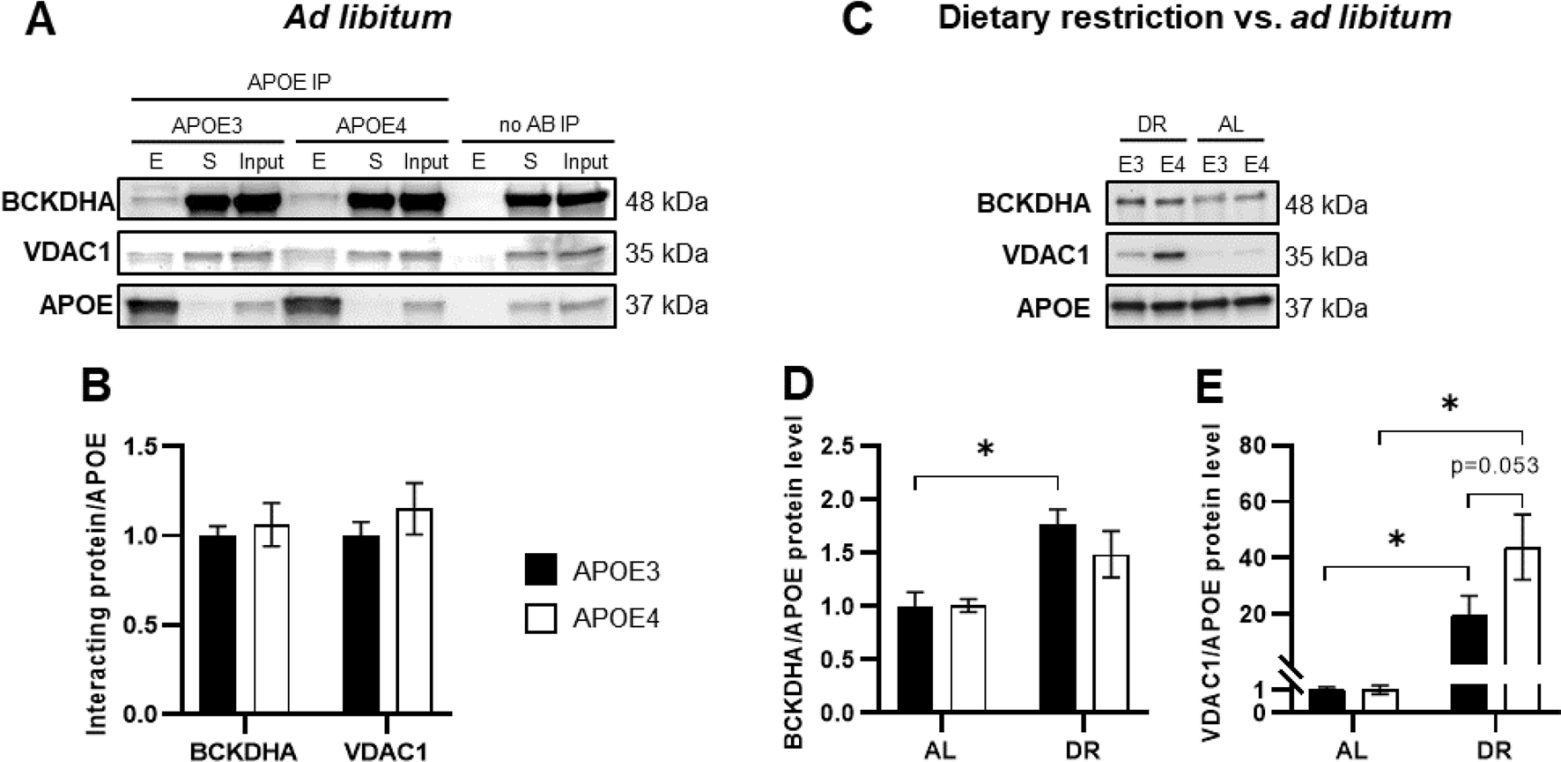
APOE-protein interactions with BCKDHA and VDAC1 in the livers of APOE3 and APOE4 mice with ad libitum (AL) or restricted (DR) access to the high-fat and high-sugar diet. **A** Representative Western blot images showing the presence of BCKDHA and VDAC1 in APOE co-IP eluates (**E**) from AL-fed APOE TR mouse livers. S, post-IP supernatant. **B** In AL-fed mice, there was no difference between the APOE3 and APOE4 groups in the APOE–BCKDHA (*p* = 0.626) and APOE–VDAC1 (*p* = 0.352) interactions (independent samples *t* test). **C** Representative Western blot images of APOE co-IP eluates showing the effect of DR on the interactions of APOE with BCKDHA and VDAC1. **D** In response to DR, the interactions with APOE were significantly increased. *(*p* < 0.05) indicates a significant difference. BCKDHA–APOE3: *p* = 0.008; BCKDHA–APOE4: *p* = 0.171 (one-way ANOVA, Šídák’s multiple comparison test). **E** VDAC1–APOE3/APOE4: *p* < 0.001 (one-way ANOVA, Fisher’s least significant difference test). Isoform-dependent differences were observed (*p* = 0.053) for the VDAC1–APOE interaction in DR-fed mice. VDAC1–APOE interaction data were log-transformed to ensure a normal distribution. Protein levels were normalized to the immunoprecipitated APOE protein level, and the results are shown relative to the APOE3 AL group (means ± SEMs, *n* = 5–6)

Representative images of APOE co-IP followed by Western blotting in liver tissue lysates from AL-fed male mice are shown in Fig. 2A. These results are in line with the previously established protein–protein interactions in cultured hepatocytes (Fig. 1). No APOE isoform-dependent effect on the interaction with BCKDHA and VDAC1 was observable in obese APOE TR mice (Fig. 2B). However, the APOE protein–protein interactions were significantly stronger upon DR (Fig. 2C–E). The VDAC1–APOE interaction was increased by 20- and 40-fold in APOE3 and APOE4 DR-fed mice, respectively (Fig. 2E), compared with their AL-fed counterparts. In addition, a trend towards an isoform-dependent difference could be observed, with an increased interaction in APOE4 mice compared with APOE3 mice (*p* = 0.053). The total protein levels of BCKDHA and VDAC1 in liver total protein lysates were not different between the groups (Fig. S3).

#### Hepatic BCKD complex activity is increased in APOE4 mice

**Fig. 3:**
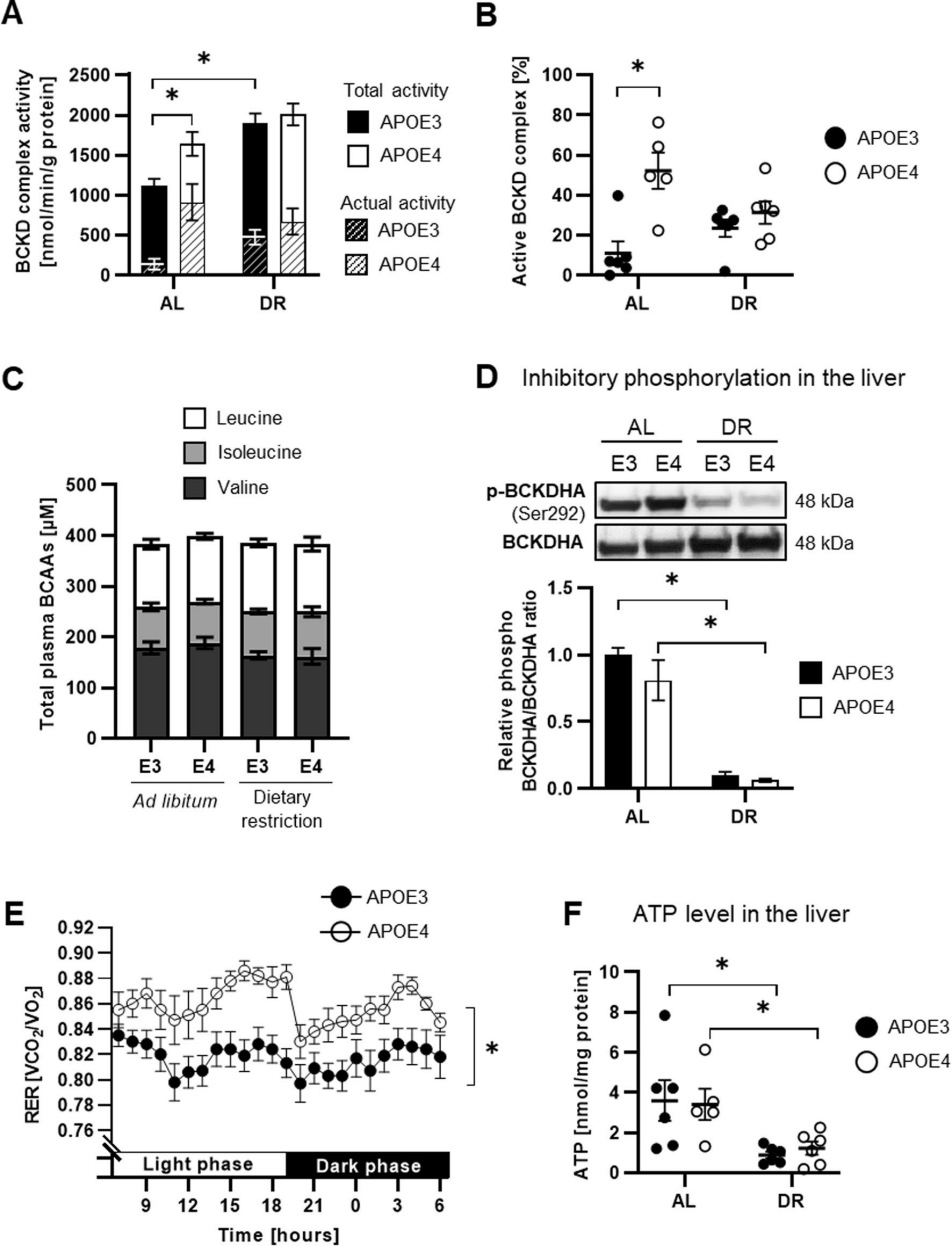
The activity and activation status of the hepatic BCKD complex are higher in obese APOE4 mice than in APOE3 mice and modulated by dietary restriction (DR). **A** Obese APOE4 mice exhibited significantly higher total BCKD activity (filled column) than APOE3 mice (*p* = 0.038). Their lean littermates had an overall higher total activity, but this difference was only significant in APOE3 mice (*p* = 0.001). The actual BCKD complex activity (striped column) is likewise higher in the AL-fed APOE4 mice than in the APOE3 mice. **B** The activity state of BCKD is calculated from the ratio of actual and total activity. The proportion of active BCKD complex in the liver was significantly higher in obese APOE4 mice than in APOE3 mice (*p* < 0.001), but the difference was not significant under DR (*p* = 0.608; two-way ANOVA, Tukey’s multiple comparison test). **C** Total plasma BCAA levels were not significantly different between the groups (one-way ANOVA; *p* = 0.948). **D** Phosphorylation of BCKDHA (Ser292) in the liver was significantly decreased under DR (both genotypes: *p* < 0.001; one-way ANOVA, Šídák’s multiple comparison test). **E** The 24-h RER was significantly higher in obese APOE4 mice than in APOE3 mice (*p* < 0.001; paired *t* test). **F** In response to DR, mice had lower hepatic ATP levels than their obese littermates (APOE3: *p* = 0.011, APOE4: *p* = 0.023; two-way ANOVA, Šídák’s multiple comparison test). A significant effect of diet (*p* = 0.001) was found by two-way ANOVA. For statistical analysis, ATP data were log-transformed to ensure homogeneity of variance. All data are shown as the means ± SEMs (*i* = 3–6). *(*p* < 0.05) indicates a significant difference between groups

BCKDHA is a component of the BCKD complex that catalyses the degradation of branched-chain alpha-keto acids (BCKAs) derived from transamination of BCAAs. The end product, acetyl-CoA, then enters the tricarboxylic acid (TCA) cycle. To investigate the potential physiological importance of the BCKDHA–APOE interaction and the effects of the APOE isoform, the enzyme activity of the BCKD complex was measured in the livers of AL- and DR-fed mice. In response to AL feeding, APOE4 mice showed significantly higher total BCKD activity than APOE3 mice (Fig. 3A). Upon DR, total enzyme activity was increased, and the increase was statistically significant in APOE3 mice. The activity state of BCKD is the ratio of actual activity before activation to total activity obtained after activation by phosphatase treatment [25]. The proportion of active BCKD complex was significantly higher (almost fivefold) in APOE4 than in APOE3 AL-fed mice (Fig. 3B). This effect was no longer evident under DR.

It has been shown that BCAA catabolism is reduced under high-fat diet feeding conditions and during the development of hepatic steatosis in mice [25]. Therefore, BCKD complex activity was correlated with liver weight in our mice. There was a highly significant negative correlation between the proportion of active BCKD complex and liver weight in AL-fed mice (*r* =  − 0.877; *p* < 0.001) (Fig. S2).

To investigate whether the altered BCKD complex activity was reflected in the levels of circulating BCAAs, the plasma concentrations of the BCAAs leucine, isoleucine, and valine were measured by HPLC. However, no significant differences in the concentrations of individual BCAAs or total BCAAs were detected between the groups (Fig. 3C).

Inhibitory phosphorylation of BCKDHA at Ser292 can be used as a marker of the BCKD complex activity status and was therefore measured by Western blotting. In response to DR, the phosphorylation of BCKDHA at Ser292 in the liver was significantly decreased compared with that in AL-fed mice, indicating increased activity of the BCKD complex (Fig. 3D). No differences between APOE3 and APOE4 mice were observed.

In contrast, BCKDHA phosphorylation in skeletal muscle showed a slight increase upon DR (Fig. S4). However, the BCKD complex is less important in skeletal muscle than in the liver [25], in contrast to branched-chain amino acid transaminase 2 (BCAT2), which is prominently expressed in muscle and catalyses the first step of BCAA degradation, transamination of BCAAs to BCKAs. Interestingly, the BCAT2 protein levels in muscle were not different between the groups (Fig. S4). The 24-h RER was significantly higher in AL-fed APOE4 mice than in APOE3 mice (Fig. 3E), indicating altered substrate utilization.

Unfortunately, enzymatic activity of the BCKD complex was not detectable in cultured hepatocytes (HepG2 and Huh7). Analysis of phosphorylated and total BCKDHA by Western blotting in APOE-transfected HepG2 cells (Fig. S4) showed no differences between the APOE3 and APOE4 groups, in line with the findings in mouse livers.

#### The mTOR pathway and ATP level are not affected by the APOE isoform

Mammalian target of rapamycin (mTOR) is activated by leucine, while leucine is also catabolized by the BCKD complex. Therefore, we further investigated whether an altered capacity for BCAA catabolism modulates mTOR signalling. The phosphorylation states of mTOR, protein 70 S6 kinase (P70S6K), protein kinase B (AKT), and AMP-activated protein kinase (AMPK) were analysed by Western blotting in the livers of male APOE TR mice (Table S7). The ratios of phosphorylated to total target protein for mTOR, P70S6K, and AKT were lower in DR-fed mice, and the difference in mTOR and AKT was statistically significant in APOE3 mice. However, the phosphorylation of AMPK, the direct counterpart of mTOR, was not different between the groups. Interestingly, the hepatic ATP level was significantly reduced in response to DR, without any apparent effect of the APOE isoform (Fig. 3F).

## Discussion

From co-IP experiments with APOE as the bait protein, BCKDHA was selected as the most promising APOE-interacting candidate because this interaction was previously detected only in an untargeted interactome study and has not been further investigated in functional studies [26]. In addition, two other subunits of the BCKD complex, namely branched-chain alpha-keto acid dehydrogenase subunit beta and dihydrolipoyl transacylase, were among the proteins detected by co-IP/LC–MS. According to the BioGRID 4.4 database, in addition to BCKDHA, three other proteins from the list of 28 potential APOE-binding proteins have been described previously to interact with APOE, including leucine-rich repeat-containing protein 59 [26], serum albumin [27], and VDAC1 [28–31]. The BCKDHA–APOE interaction was enhanced upon DR in APOE TR mice but was not different between the APOE3 and APOE4 groups in either mouse livers or cultured hepatocytes. However, the proportion of active BCKD enzyme complex, which is a measure of the BCKD activity status, was greatly increased in AL-fed APOE4 mice compared with APOE3 mice. This finding is of special importance, as it indicates a higher hepatic BCKA catabolic rate in obese APOE4 mice than in APOE3 mice.

However, because the BCKDHA–APOE interaction was not altered depending on the APOE isoform, it may be that the higher BCKD complex activity observed in APOE4 mice occurs independently of the BCKDHA–APOE interaction. Instead, broader physiological effects of APOE could cause the changes in BCKD activity. One physiological parameter in which APOE3 and APOE4 differ is the development of body weight on a high-energy diet [19, 32, 33], with APOE3 mice being more prone to diet-induced obesity. At the same time, impaired BCAA metabolism is observed in obesity and diabetes, and circulating BCAA levels are associated with worsening metabolic health and are higher in obese individuals [34, 35]. A higher BCAA catabolic rate may thus counteract obesity-related metabolic alterations or, conversely, a lower body weight could positively affect the BCAA metabolism [36]. Therefore, the higher BCKD activity in APOE4 mice may also be related to the lower body weight gain and liver weight in these mice. When fed a HFSD, APOE3 mice exhibited more pronounced hepatic steatosis than APOE4 mice (manuscript in preparation, Huebbe et al. 2023). We therefore plotted individual liver weights and BCKD activities and observed a significant inverse relationship. This correlation was less significant for body weight, indicating that instead of obesity in general, the higher liver mass in APOE3 mice—particularly hepatic steatosis—impairs BCKA catabolism. This is in line with studies showing a correlation between decreased BCAA catabolism and fatty liver [37, 38].

No difference was found in total BCKDHA protein concentrations between groups, although DR-fed mice showed higher total BCKD activity. However, the BCKDHA is only one out of many subunits of the BCKD complex, and thus, the protein concentration of the BCKDHA may not directly predict the whole complex activity.

The DR-fed mice showed a higher total BCKD activity along with a relatively small proportion of it being actually active. However, it needs to be considered that the DR-fed mice were subjected to a longer starvation period than the AL-fed mice. Four hours before sacrifice, the food was taken away from all groups. At this point, the mice under DR had already fasted for several hours, as they ate their food almost completely immediately after the feeding time. Starvation has been shown to alter the activity of the BCKD complex, with the length of restriction also affecting activity [39]. For example, a significant increase in total BCKD complex activity was observed in the liver of rats fasted for 3 days [40]. Therefore, the differences in fasting time may contribute to the diet-dependent altered complex activity.

Previous studies have shown that BCKD activity is decreased in subcutaneous adipose tissue in severe obesity [41] and the levels of several genes involved in BCAA catabolism are decreased in the liver and white adipose tissue of *ob/ob* mice compared with those of wild-type mice [35]. Interestingly, despite the higher hepatic catabolic rate, fasting circulating BCAA levels were not altered in APOE4 mice. Because other tissues contribute significantly to BCAA catabolism, such as skeletal muscle [25], it is conceivable that the effect of the liver on plasma BCAAs is equalized in APOE4 mice. Furthermore, plasma BCAA levels respond to food deprivation and change postprandially in a time-dependent manner [35, 42]. Our mice were fasted for at least four hours before sample collection, which may have impacted circulating BCAAs, possibly blunting any potential differences between APOE3 and APOE4 mice. The finding that the BCAA concentration was similar not only between APOE3 and APOE4 mice but also between lean DR and obese AL-fed mice substantiates this assumption. Therefore, further studies are needed to validate the hypothesis that circulating BCAA levels are dynamically modulated dependent on the APOE isoform in obese mice. To date, no studies have systematically investigated the involvement of APOE in amino acid metabolism. However, elevated isoleucine levels have been found in human *APOEε4* carriers [43]. It remains unclear whether this increase is related to APOE isoform-dependent modulation of BCAA catabolism.

Modulation of BCAA catabolism can affect other energy substrates, such as fatty acids. Mice with genetically impaired BCAA catabolism had increased fat accumulation in muscle [44], whereas activation of the BCKD complex is associated with increased fatty acid oxidation in the liver [45]. Notably, we observed APOE isoform-dependent differences in the RER in our mice, with a significant increase in APOE4 mice. We already suggested an APOE isoform-dependent shift in substrate utilization in a previous study [19]. Evidence has been presented for increased fatty acid oxidation in the muscle in the presence of APOE4, in line with the higher BCKD complex activation status in APOE4 mice observed in the present study and the results of White et al. [45], who related increased fatty oxidation to higher BCKD activity. However, increased fatty acid oxidation and BCAA catabolism may not explain the higher RER observed in APOE4 mice (0.86 versus 0.82 in APOE3 mice).

VDAC1 is involved in multiple mitochondrial processes and plays a central role in mitochondrial metabolism [46]. Therefore, the interaction of APOE with VDAC1 and the significant increase in this interaction in response to DR are highly important findings. VDAC1 is a known gatekeeper for the exchange of molecules such as ATP, NADPH, calcium, and reactive oxygen species (ROS) through the OMM [46]. DR has been suggested to be associated with altered ROS production, reduced oxidative damage, and changes in mitochondrial respiration capacity [47]. The strengthening of the VDAC1–APOE interaction in DR-fed mice may point to a regulatory role in molecule exchange through the OMM and thus in mitochondrial function.

The interaction of VDAC1 and APOE has been shown previously in interactome studies conducted in HEK293T cells [28–30], and recombinant human APOE has been described to interact with VDAC1 in cultured rat cardiomyocytes [31]. The strength of the VDAC1–APOE interaction and the respiratory capacity did not differ in an APOE isoform-dependent manner [31]. However, it has been suggested that mitochondrial permeability is impaired due to opening of the mitochondrial permeability transition pore (mPTP), resulting in mitochondrial swelling and dysfunction [31]. In contrast, caloric restriction in mice hinders mPTP opening due to an increase in the mitochondrial calcium buffering capacity [48]. VDAC1 is a component of the mPTP [49], which suggests that APOE is also involved in this pore formation. It may be further speculated that the stronger VDAC1–APOE interaction induced by DR stabilizes the mPTP, while the weaker interaction in obese mice contributes to mPTP opening and thus promotes mitochondrial dysfunction. Since both calcium and ROS homeostasis and the *APOE* gene are associated with the development of age-related diseases [2, 50], there is a particular need for research to identify possible links and to further characterize the significance of the VDAC1–APOE interaction in this context.

To interact with mitochondrial proteins, APOE needs to enter the mitochondrion, at least for interactions with mitochondrial matrix proteins such as the BCKDHA. Because APOE is known to be a secretory protein and does not contain a mitochondrial signal peptide, it is unclear how APOE crosses the mitochondrial membranes. Nothing is known about a potential mitochondrial translocation mechanism for APOE so far, although several studies have already shown the localization of the APOE protein in mitochondria [52–54]. A detailed review on mitochondrial localization and function of APOE is given in our review (Rueter et al. [55]).

Additional evidence of the importance of APOE in mitochondria and mitochondrial function has been provided by studies in mice with genetic deficiency of *Apoe*. The hepatic expression of mitochondrial proteins, including ATP synthase subunit beta and peroxiredoxin 3, which are involved in ATP synthesis and ROS detoxification, respectively, was altered in the absence of endogenous *Apoe* [51].

Overall, the present study is limited by its model, i.e. mice genetically modified to express human APOE variants that interact with endogenous proteins. It may be questioned whether the investigated protein–protein interactions occur only in these targeted replacement mice, and the relevance to humans needs to be established. However, we were able to verify the APOE-protein interactions with human proteins in cultured human hepatocytes. In addition, the murine and human BCKDHA and VDAC1 proteins show a high degree of amino acid sequence similarity (BCKDHA > 92%; VDAC1 > 98%), suggesting a high probability that the potential APOE-binding sites are identical. APOE TR mice are a valuable model for studying phenotypic, metabolic, and molecular changes in response to APOE isoforms that is applicable to humans, as has been demonstrated in the past [4, 7].

BCAAs are important signalling molecules in health and metabolic disease, and their circulating levels are elevated in type 2 diabetes, obesity [35], and nonalcoholic fatty liver disease [38]. Therefore, the interaction of APOE with hepatic BCKDHA and BCAA catabolism established here may be of special clinical relevance, especially considering the higher enzyme activity associated with APOE4, which could prevent the accumulation of BCAAs. However, whether the elevated BCAA levels in diabetic patients are the cause or consequence of the disease is not fully understood [36]. Targeting the APOE–BCKD complex interaction could represent a novel approach for metabolic disease therapy and prevention.

## Conclusion

In conclusion, the results of the present work highlight the relevance of APOE to hepatic mitochondrial function and energy metabolism. A novel APOE-interacting protein, BCKDHA, was identified, and its influence on BCKD activity in response to diet-induced obesity and dietary restriction was presented. Further research is needed to evaluate the role of APOE in BCAA catabolism, including studies in *Apoe* knockout models, and to explore the underlying molecular mechanisms of the interaction, such as the identification of the involved binding sites. Moreover, the transport mechanisms by which APOE may pass through mitochondrial membranes and enter the inner matrix to interact with the BCKD complex remain to be elucidated.

### Supplementary Information

Below is the link to the electronic supplementary material.

Supplementary file1 (DOCX 40 KB)

Supplementary file2 (DOCX 206 KB)

Supplementary file3 (XLSX 164 KB)

## Data Availability

LC–MS data have been deposited to the ProteomeXchange Consortium via the PRIDE partner repository with the dataset identifier PXD033961.

## Abbreviations

ACN: Acetonitrile
AKT: Protein kinase B
AL: Ad libitum
AMPK: AMP-activated protein kinase
APOE: Apolipoprotein E
BCAA: Branched-chain amino acid
BCAT2: Branched-chain amino acid transaminase 2
BCKA: Branched-chain alpha-keto acids
BCKDHA: Branched-chain alpha-keto acid dehydrogenase subunit alpha
BT2: 3,6-Dichloro-benzo[b]thiophene-2-carboxylic acid
Co-IP: Coimmunoprecipitation
DR: Dietary restriction
FA: Formic acid
FBS: Foetal bovine serum
HFSD: High-fat high-sugar diet
HH3: Histone H3
LC–MS: Liquid chromatography–mass spectrometry
LDL: Low-density lipoprotein
mPTP: Mitochondrial permeability transition pore
mTOR: Mammalian target of rapamycin
OMM: Outer mitochondrial membrane
P70S6K: Protein 70 S6 kinase
PLA: Proximity ligation assay
RER: Respiratory exchange ratio
ROS: Reactive oxygen species
TCA: Tricarboxylic acid cycle
TR: Targeted replacement
VDAC1: Voltage-dependent anion-selective channel 1
