# Supplementary material for: The mitochondrial BCKD complex interacts with hepatic apolipoprotein E in cultured cells in vitro and mouse livers in vivo
Source: Cell Mol Life Sci. 2023 Feb 7;80(3):59. doi: 10.1007/s00018-023-04706-x (PMC9905200; doi:10.1007/s00018-023-04706-x)
Supplement: Supplementary file 1 — Supplementary file1 (DOCX 40 KB) [file 18_2023_4706_MOESM1_ESM.docx]

**Supplementary Material & Methods**

**Title:** The mitochondrial BCKD complex interacts with hepatic apolipoprotein E in cultured cells *in vitro* and mouse livers *in vivo*

**Journal:** *Cellular and Molecular Life Sciences*

**Authors:** Johanna Rueter, Gerald Rimbach, Christian Treitz, Anke Schloesser, Kai Luersen, Andreas Tholey, Patricia Huebbe

**Corresponding author:** Gerald Rimbach; Hermann-Rodewald-Strasse 6, D-24118 Kiel, Germany; Tel.: +49 431 880 2583; Fax: +49 431 880 2628; Mail: rimbach@foodsci.uni‑kiel.de

Table S1: Compositions of buffers and solutions.

| **Assay** | **Buffer** | **Composition** |
| --- | --- | --- |
| **Amino acid analysis, HPLC** | **Borate buffer** | 0.4 M boric acid  0.4 M potassium chloride  0.4 M sodium hydroxide  pH 9.9 |
|  | **Derivatization reagent** | 5 mg OPA in 500 µl ddH_2_O  1 ml 0.4 M borate buffer  27 µl ethanethiol |
|  | **Mobile phase buffer A** | 60 mM sodium acetate  0.6% tetrahydrofuran  pH 8.0 |
|  | **Mobile phase buffer B** | 700 ml acetonitrile  200 ml 0.1 M sodium acetate  50 ml methanol |
| **ATP lysis buffer** | | 25 mM Tris  4 mM EDTA  0.5% (w/v) Triton X-100  pH 7.5 |
| **BCKD assay** | **Assay buffer 2x** | 60 mM potassium phosphate  0.8 mM thiamine pyrophosphate  0.8 mM coenzyme A  2 mM NAD+  4 mM DTT  2% (w/v) Triton X-100  4 mM magnesium chloride  10 units/ml dihydrolipoamide dehydrogenase (added immediately before measurement)  pH 7.4 at 30 °C |
|  | **Enzyme diluent** | 0.54 mM riboflavin 5′-monophosphate sodium salt hydrate  200 mM Tris  294 mM potassium chloride  0.025% (w/v) BSA  pH 7.5 |
|  | **Suspending buffer** | 25 mM HEPES  0.2 mM EDTA  1 mM DTT  0.4 mM thiamine pyrophosphate  50 mM potassium chloride  0.02 mg/ml leupeptin  0.1% (w/v) Triton X-100  pH 7.4 at 37 °C |
|  | **Extraction buffer** | 50 mM HEPES  2 mM EDTA  5 mM DTT  0.5 mM thiamine pyrophosphate  0.5 mM (S)-CPP sodium salt  50 mM potassium fluoride  3% (w/v) Triton X-100  2% (v/v) bovine serum  pH 7.4 at 4 °C |
| **Co-IP,  Western blot analysis** | **NP40 lysis buffer** | 150 mM sodium chloride  50 mM Tris  5 mM EDTA  1% NP40  pH 7.4  Added before use:  1:100 protease inhibitor cocktail (Sigma, Germany)  1:200 200 mM phenylmethylsulfonyl fluoride  1:10 PhosSTOP (Roche, Germany) |
|  | **Tris-buffered saline/Tween20 (TBS/T)** | 50 mM Tris  150 mM sodium chloride  0.05% Tween 20  pH 7.5 |
|  | **Western blot transfer buffer** | 200 ml Trans-Blot Turbo 5X Transfer Buffer  200 ml ethanol  600 ml ddH_2_O |

Table S2: Primary and secondary antibodies used for Western blot analysis and proximity ligation assays.

| **Name** | **Supplier** | **Cat no.** | **Clone no.** |
| --- | --- | --- | --- |
| AMP-activated protein kinase | Santa Cruz | sc-25792 | Polyclonal |
| Phospho-AMP-activated protein kinase (Thr172) | Cell signaling | 2535 | 40H9 |
| Apolipoprotein E | Santa Cruz | sc-13521 | A1.4 |
| Apolipoprotein E | Santa Cruz | sc-390925 | F-9 |
| Branched-chain alpha-keto acid dehydrogenase E1 subunit alpha | Abclonal | A9806 | Polyclonal |
|  | Novus Biologicals | NBP2-15552 | Polyclonal |
| Phospho-branched-chain alpha-keto acid dehydrogenase E1 subunit alpha (Ser292) | Bethyl laboratories | A304-672A-M | Polyclonal |
| Branched-chain amino acid transaminase 2 | Proteintech | 16417-1-AP | Polyclonal |
| Histone H3 | Novus Biologicals | NBP2-36468SS | 1B1-B2 |
| Mammalian target of rapamycin | Cell signaling | 4517S | L27D4 |
| Phospho-mammalian target of rapamycin (Ser2448) | Cell signaling | 5536S | D9C2 |
| Protein 70 S6 kinase | Cell signaling | 2708S | 49D7 |
| Phospho-protein 70 S6 kinase (Thr389) | Cell signaling | 108D2 | 108D2 |
| Protein kinase B | Cell signaling | 4691 | C67E7 |
| Phopsho-protein kinase B (Ser473) | Cell signaling | 4060 | D9E |
| Voltage-dependent anion-selective channel protein 1 | Proteintech | 55259-1-AP | Polyclonal |
|  | Santa Cruz | sc-8828 | Polyclonal |
| Duolink in situ PLA probe anti-mouse MINUS | Sigma | DUO92004 |  |
| Duolink in situ PLA probe anti-mouse PLUS | Sigma | DUO92001 |  |
| Duolink in situ PLA probe anti-rabbit PLUS | Sigma | DUO92002 |  |
| Immun-Star goat anti-mouse (GAM)-HRP conjugate | Bio-Rad | 1705047 |  |
| Immun-Star goat anti-rabbit (GAR)-HRP conjugate | Bio-Rad | 1705046 |  |
| Rabbit anti-goat IgG (H+L)-HRP conjugate | Bio-Rad | 1721034 |  |

Table S3 Adjusted HPLC gradient conditions.

Buffer A: 60 mM sodium acetate, 0.6% tetrahydrofuran; Buffer B: acetonitrile, 0.1 M sodium acetate, methanol; 14:4:1.

| **Time [min]** | **% B** | **Flow [ml/min]** |
| --- | --- | --- |
| 0.00 | 20.0 | 0.80 |
| 6.00 | 30.0 | 0.80 |
| 9.00 | 33.5 | 0.80 |
| 14.00 | 35.0 | 0.80 |
| 17.00 | 38.0 | 0.80 |
| 27.00 | 38.0 | 1.30 |
| 40.00 | 100.0 | 1.30 |
| 47.00 | 100.0 | 0.80 |
| 48.00 | 20.0 | 0.80 |
| 54.00 | 20.0 | 0.80 |

Table S4: Antibody pairs used for PLA controls and experiments.

For each PLA experiment, two primary antibodies against the two target proteins are needed, and these antibodies must be raised in different species. Secondary antibodies conjugated to oligonucleotides are called PLA probes. The PLA probes must be selected according to the species of the primary antibodies, and one MINUS and one PLUS PLA probe per experiment is needed. m, primary anti-mouse; r, primary anti-rabbit.

| **PLA experiments** | | **Primary antibodies** | **PLA probes** |
| --- | --- | --- | --- |
| **Interactions of interest** | **BCKDHA−APOE** | BCKDHA (r, NBP2-15552) APOE (m, sc-13521) | Mouse MINUS  Rabbit PLUS |
|  | **VDAC1−APOE** | VDAC1 (r, 55259-1-AP) APOE (m, sc-13521) | Mouse MINUS  Rabbit PLUS |
| **Controls** | **APOE positive control** ‘single recognition PLA’ | APOE (m, sc-13521) | Mouse MINUS  Mouse PLUS |
|  | **APOE negative control** ‘technical control’ | APOE (m, sc-13521) | Mouse MINUS  Rabbit PLUS |
|  | **BCKDHA−HH3** ‘biological control’ | BCKDHA (r, NBP2-15552) HH3 (m, NBP2-36468SS) | Mouse MINUS  rabbit PLUS |
|  | **No AB** ‘technical control’ | - | Mouse MINUS  Rabbit PLUS |

**Coimmunoprecipitation**

*Co-IP/LC−MS*

For LC−MS analysis, the final washing steps were performed with decreasing NP40 concentrations (0.5-0.05%) and additional wash steps were performed with ammonium bicarbonate buffer (50 mM, pH 8). Proteins were eluted from the beads with 0.5% formic acid (FA) for 10 min at room temperature, and the eluates were stored at -80 °C until use.

Briefly, the co-IP eluates were lyophilized to dryness, resuspended in 20 µl of 50 mM triethylammonium bicarbonate buffer, reduced with 12 mM tris(2‑carboxyethyl)phosphine (1 h, 60 °C), alkylated with 40 mM chloroacetamide (30 min, 20 °C), and digested with 0.5 µg of trypsin (overnight, 37 °C).

Samples were analysed on a Dionex Ultimate 3000 nano-UHPLC system coupled to a QExactive Plus MS system (Thermo Fisher Scientific, Bremen, Germany). Samples were concentrated and washed for 5 min (Acclaim PepMap 100 C18, 10 mm × 300 μm, 3 μm, 100 Å; Thermo Fisher Scientific, Dreieich, Germany) with 3% acetonitrile (ACN) and 0.1% trifluoroacetic acid at a flow rate of 30 µl/min for 2 min prior to peptide separation using an Acclaim PepMap 100 C18 analytical column (50 cm × 75 μm, 3 μm, 100 Å; Thermo Fisher Scientific, Dreieich, Germany). Eluent A (0.05% FA) and eluent B (80% ACN, 0.04% FA) were used at a flow rate of 300 nl/min for separation with a gradient from 5% to 50% eluent B over 120 min, followed by an increase to 95% B over 5 min and 10 min of isocratic elution at 95% B. The spray voltage applied on a metal-coated PicoTip Emitter (10-μm tip size; New Objective, Woburn, MA, USA) was 1.6 kV, with a source temperature of 250 °C. Full-scan MS spectra were acquired between 350 and 2000 m/z at a resolution of 70,000 at m/z 400. The 10 most intense precursors with a charge state >2+ were selected with an isolation window of 3.0 m/z and fragmented by higher-energy collisional dissociation with a normalized collision energy of 25 and a resolution of 17,500. Lock mass (445.120025) and dynamic exclusion (15 s) were enabled.

The raw MS files were processed with Proteome Discoverer 2.2.0.388 (Thermo Fisher Scientific, Bremen, Germany). MS/MS spectra were searched using the SEQUEST search algorithm against a database containing common contaminants and against the canonical murine and human databases (52,015 and 71,591 entries, respectively; UniProt Sept 2017). The data were searched with tryptic protease specificity allowing 2 missed cleavages. An MS1 tolerance of 15 ppm and an MS2 tolerance of 0.02 Da were implemented. Oxidation (15.995 Da) of methionine residues was set as a variable modification, along with protein N-terminal acetylation (42.011 Da), while carbamidomethylation of cysteine residues was set as a static modification (57.021 Da). Peptide spectrum matches were filtered to a false discovery rate (FDR) of less than 1%, and protein group identifications were filtered to an FDR of less than 1%. Label-free quantification was performed using a Minora feature detector. The complete list of identified protein groups is provided in Table S5.

The over 300 identified proteins were filtered according to the following criteria: All antibody fragments and chains were removed from the list, since also endogenous murine antibodies bind to the Sepharose beads, not only the APOE antibody used for the co-IP. In addition, only proteins with at least two identified peptides were considered for inclusion in the final list. To minimize possible artefacts due to nonspecific binding and contaminants, the ratio of the abundance of the detected proteins in the APOE co-IP samples to the respective noAB IP control was calculated. Only proteins that showed twofold or higher enrichment in at least two of the four co-IPs and fivefold or higher enrichment in at least one co-IP compared with the noAB control were included in the final list.

LC‒MS data have been deposited to the ProteomeXchange Consortium [1] via the PRIDE partner repository with the dataset identifier PXD033961.

*Co-IP/Western blot analysis*

For Western blot analysis after co-IP, bound proteins were eluted with 15 µl of reducing protein gel loading buffer (ROTI Load 1, Roth, Karlsruhe, Germany) for 5 min at 95 °C. The beads were briefly sedimented by centrifugation, and the supernatant was collected. The elution process was repeated, and the two supernatants were pooled before loading onto gels for Western blotting. After the identification of potential APOE-binding proteins, reverse co‑IP using IP antibodies against the corresponding target proteins was carried out according to the same protocol.

**Proximity ligation assay**

Huh7 cells were seeded in 24-well plates on cover slips coated with poly-L-lysine for 20 min. Twenty-four hours after seeding, the cells were transfected, and after 24 hours, the cells were incubated for 30 min at 37 °C to stain mitochondria. Fixation of the cells was carried out using 4% paraformaldehyde in DPBS and incubation for 15 min at room temperature. The cells were washed three times with DPBS and permeabilized with DPBS containing 0.1% NP40 and 100 mM glycine for 30 min at room temperature.

PLA was performed according to the manufacturer's instructions using Duolink *In Situ* Detection Reagents Green, wash buffers, and appropriate combinations of mouse and rabbit PLA probes. All Duolink and PLA reagents were purchased from Sigma (Steinheim, Germany). For the antibody combinations and the corresponding PLA probes, see Table S4. After permeabilization, the cells were blocked with Duolink blocking solution for 60 min at 37 °C. Primary antibodies were diluted in Duolink antibody diluent and incubated for 60 min at room temperature. PLA probes were diluted in Duolink antibody diluent, applied to the cover slips, and incubated for 60 min at 37 °C. After incubation with ligase (30 min, 37 °C) and then polymerase (100 min, 37 °C), a fluorescent green-labelled circular DNA strand is formed and amplified if the two target proteins are in spatial proximity to each other (<40 nm). For detection of PLA signals in APOE-transfected cells, the polymerase incubation time was reduced to 50 min because overexpression of APOE caused the PLA signals to overlap, and it was not possible to quantify these signals.

The cover slips were repeatedly washed with Duolink wash buffer A or B between the individual incubation steps. In the last step, the samples were mounted with Duolink *In Situ* Mounting Medium with DAPI onto slides and sealed with nail polish after 15 min. PLA signals were detected using an inverted fluorescence microscope (Zeiss Axio Observer D1), and AxioVision software version 4.8 (both from Carl Zeiss Microscopy, Jena, Germany) was used to acquire images. The images were analysed and edited with ImageJ 1.8.0 [2] for analysis, the PLA images were converted into binary black and white images, and the particles per cell were counted and expressed as PLA signals per nucleus. The PLA signals from at least 100 cells per sample from three independent experiments were quantified with ImageJ.

References

[1] J.A. Vizcaíno, E.W. Deutsch, R. Wang, A. Csordas, F. Reisinger, D. Ríos, et al., ProteomeXchange provides globally coordinated proteomics data submission and dissemination, Nature Biotechnology 32 (3) (2014) 223–226. https://doi.org/10.1038/nbt.2839.

[2] C.A. Schneider, W.S. Rasband, K.W. Eliceiri, NIH Image to ImageJ: 25 years of image analysis, Nature Methods 9 (7) (2012) 671–675. https://doi.org/10.1038/nmeth.2089.
