# Supplementary material for: The mitochondrial BCKD complex interacts with hepatic apolipoprotein E in cultured cells in vitro and mouse livers in vivo
Source: Cell Mol Life Sci. 2023 Feb 7;80(3):59. doi: 10.1007/s00018-023-04706-x (PMC9905200; doi:10.1007/s00018-023-04706-x)
Supplement: Supplementary file 2 — Supplementary file2 (DOCX 206 KB) [file 18_2023_4706_MOESM2_ESM.docx]

**Supplementary Tables & Figures**

**Title:** The mitochondrial BCKD complex interacts with hepatic apolipoprotein E in cultured cells *in vitro* and mouse livers *in vivo*

**Journal:** *Cellular and Molecular Life Sciences*

**Authors:** Johanna Rueter, Gerald Rimbach, Christian Treitz, Anke Schloesser, Kai Luersen, Andreas Tholey, Patricia Huebbe

**Corresponding author:** Gerald Rimbach; Hermann-Rodewald-Strasse 6, D-24118 Kiel, Germany; Tel.: +49 431 880 2583; Fax: +49 431 880 2628; Mail: rimbach@foodsci.uni‑kiel.de

Table S6: Top 28 APOE-associated proteins.

The 28 proteins identified by APOE co-IP followed by LC−MS in the livers of female APOE TR mice included 12 proteins found with high confidence in mitochondria (underlined), according to GeneCards (high confidence=4-5, on a scale of 0-5; The Human Gene Database; https://www.genecards.org/).

| **Acaa1b** | 3-ketoacyl-CoA thiolase B, peroxisomal |
| --- | --- |
| **Top 29 APOE-associated proteins** | |
| **LONP1** | Lon protease homolog, mitochondrial |
| **VDAC1** | Voltage-dependent anion-selective channel protein 1 |
| **DBT** | Lipoamide acyltransferase component of branched-chain alpha-keto acid dehydrogenase complex, mitochondrial |
| **BCKDHA** | 2-oxoisovalerate dehydrogenase subunit alpha, mitochondrial |
| **ACAA1A** | 3-ketoacyl-CoA thiolase A, peroxisomal |
| **LRRC59** | Leucine-rich repeat-containing protein 59 |
| **FASN** | Fatty acid synthase |
| **GLUL** | Glutamine synthetase |
| **MAT1A** | S-adenosylmethionine synthase isoform type-1 |
| **CPSF6** | Cleavage and polyadenylation-specificity factor subunit 6 |
| **BCKDHB** | 2-oxoisovalerate dehydrogenase subunit beta, mitochondrial |
| **ALDH4A1** | Delta-1-pyrroline-5-carboxylate dehydrogenase, mitochondrial |
| **CLUH** | Clustered mitochondria protein homolog |
| **RPL26** | 60S ribosomal protein L26 |
| **STIM1** | Stromal interaction molecule 1 |
| **RPL37A** | 60S ribosomal protein L37a |
| **LDHA** | L-lactate dehydrogenase |
| **VDAC2** | Voltage-dependent anion-selective channel protein 2 |
| **ACSM1** | Acyl-coenzyme A synthetase, mitochondrial |
| **GMPPA** | Mannose-1-phosphate guanyltransferase alpha |
| **SELENBP1** | Selenium-binding protein 1 |
| **PAH** | Phenylalanine-4-hydroxylase |
| **UGT1A1** | UDP-glucuronosyltransferase 1-1 |
| **FMO5** | Dimethylaniline monooxygenase [N-oxide-forming] 5 |
| **PGM2** | Phosphoglucomutase-1 |
| **ALB** | Serum albumin |
| **ALDH1B1** | Aldehyde dehydrogenase X, mitochondrial |
| **CBR1** | Carbonyl reductase [NADPH] 1 |

Table S7: Densitometric analysis of protein bands from Western blot analysis indicating the ratios of phosphorylated to total protein for AKT, mTOR, P70S6K, and AMPK in the livers of male APOE TR mice.

The ratios of phosphorylated to total protein for AKT, mTOR, and P70S6K were lower in DR than in AL-fed mice, but the differences were statistically significant only in APOE3 mice for AKT and mTOR. AKT, mTOR, P70S6K: one-way ANOVA, Šídák's multiple comparison test. AMPK: Kruskal‒Wallis test, Dunn's multiple comparison test. The data are shown as the means ± SEMs relative to the APOE3 AL group (n=5-6). * (p<0.05) indicates a significant difference between the AL and DR groups.

| Phosphorylated to total protein ratio | **APOE3** | | **APOE4** | |
| --- | --- | --- | --- | --- |
|  | *Ad libitum* | Dietary restriction | *Ad libitum* | Dietary restriction |
| **AKT** | **1.00**±0.12 | **0.48**±0.07 * (p=0.027) | **0.98**±0.14 | **0.61**±0.15 |
| **mTOR** | **1.00**±0.12 | **0.49**±0.05 * (p=0.004) | **0.85**±0.11 | **0.59**±0.06 |
| **P70S6K** | **1.00**±0.21 | **0.55**±0.07 | **0.87**±0.21 | **0.58**±0.21 |
| **AMPK** | **1.00**±0.07 | **1.05**±0.23 | **1.32**±0.18 | **0.90**±0.08 |


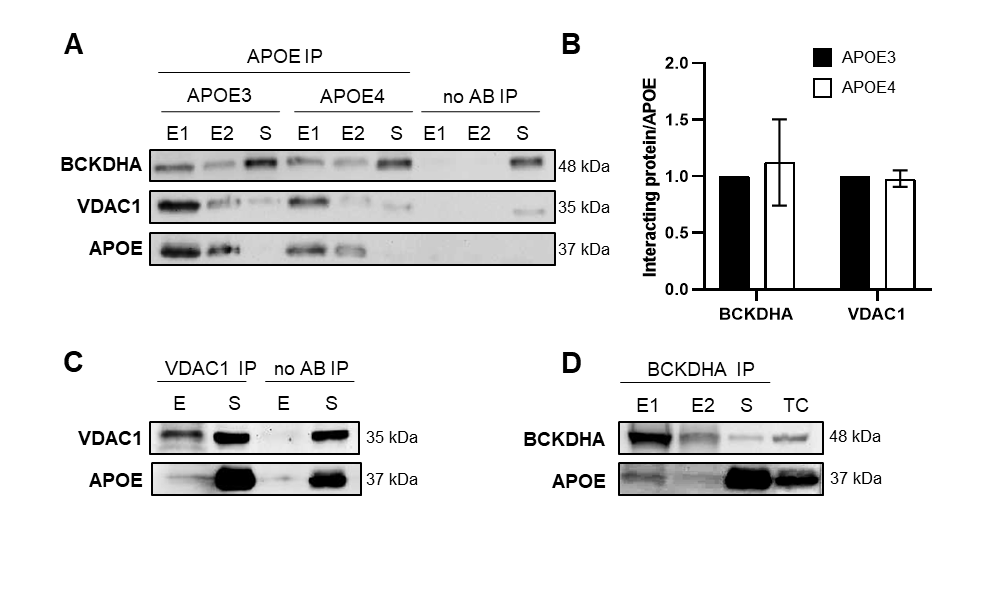


Fig. S1: APOE co-IP/Western blot analysis confirmed the LC‒MS data indicating the interactions of APOE with BCKDHA and VDAC1 in APOE-transfected Huh7 cells.

(**A**) Targeted detection of potential APOE binding proteins by Western blot analysis in APOE co-IP eluates from APOE-transfected Huh7 cells. (**B**) No APOE isoform-dependent difference in the interaction with BCKDHA (p=0.779) or VDAC1 (p=0.797) was found (one-sample t test). The amounts of the detected binding partners were normalized to the corresponding amounts of immunoprecipitated APOE protein and are indicated relative to APOE3. The data are shown relative to APOE3 as the means ± SEMs (n=3). (**C**) and (**D**) The interactions of APOE with VDAC1 and BCKDHA were confirmed by reverse co-IP in Huh7 cells. APOE was detected in the eluates (E, E1, E2) of BCKDHA and VDAC1 co-IP samples. No AB, no-antibody control; S, post-IP supernatant; TC, total protein control.


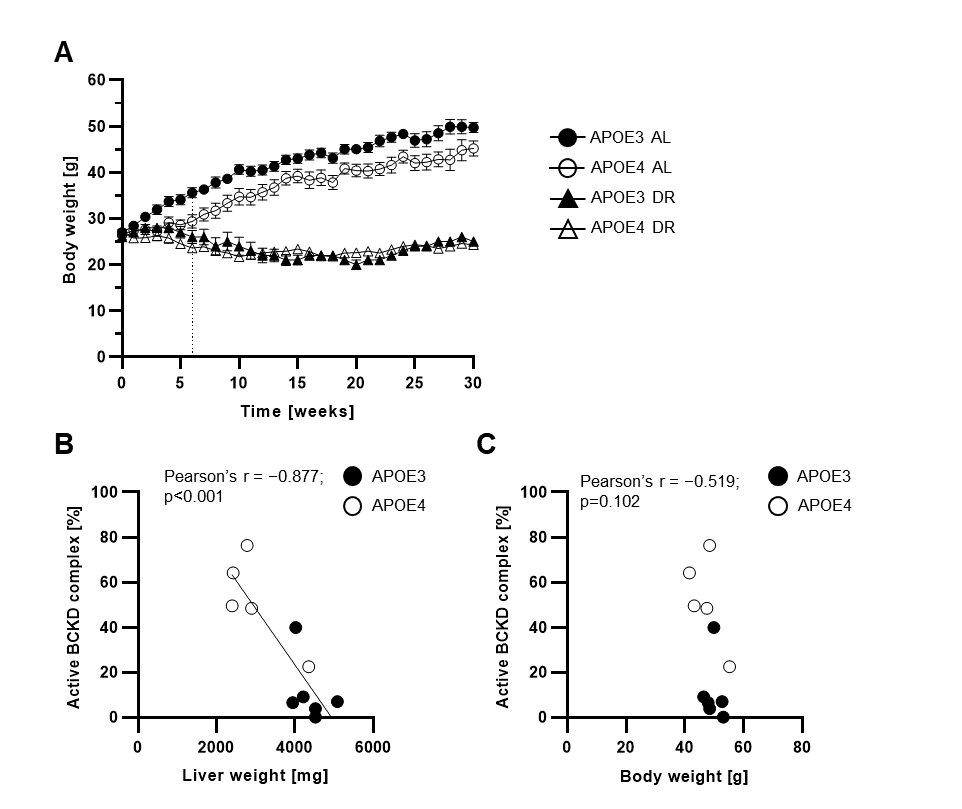


Fig. S2: Body weight gain of DR- and AL-fed male APOE TR mice and correlation of BCKD activity with liver and body weights.

(**A**) The dotted line marks the start of 30% DR after gradual implementation of DR in the first six weeks. Body weight gain was significantly different between AL and DR mice of both genotypes (p<0.001), as well as between AL-fed APOE3 mice and AL-fed APOE4 mice (p<0.001; repeated measures ANOVA, Šídák's multiple comparison test). The data are shown as the means (n=4-6). (**B**) BCKD complex activity and liver weight were highly negatively correlated in AL-fed mice (p<0.001, Pearson’s r=−0.877). (**C**) No significant correlation was found between BCKD complex activity and body weight (p=0.102, Pearson’s r=−0.519).


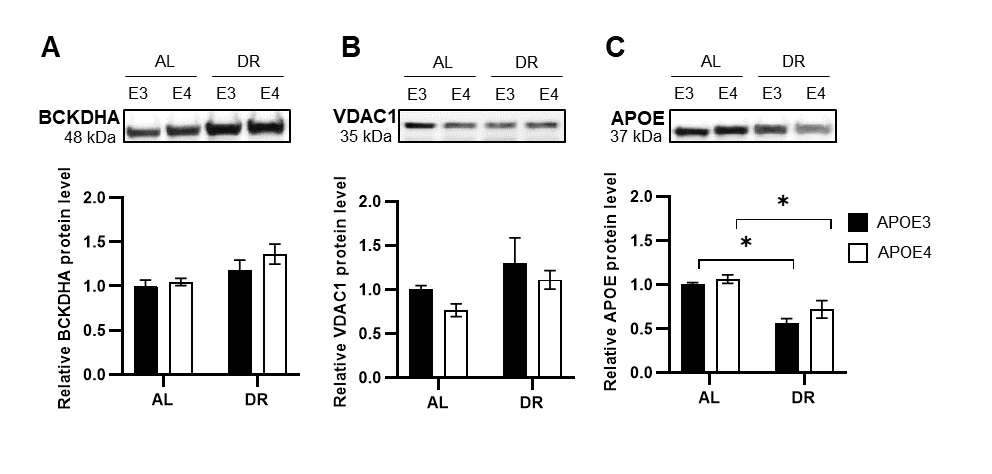


**Fig. S3: Protein levels of BCKDHA, VDAC1, and APOE in liver total protein lysates from male APOE TR mice.**

To examine whether the observed differences in protein−protein interactions may be related to altered expression of the corresponding proteins alone, Western blot analysis was performed with total protein lysates. (A) BCKDHA and (B) VDAC1 levels were not significantly affected by DR or APOE isoform. (C) However, DR mice showed lower APOE protein expression than AL-fed mice (APOE3: p<0.001; APOE4: p=0.007). The data are shown relative to APOE3 AL-fed mice (means ± SEMs, n=5-6). For statistical analysis, the Kruskal‒Wallis test with Dunn's multiple comparison test (BCKDHA), Welch’s ANOVA with Dunnett's T3 multiple comparison test (VDAC1) and one-way ANOVA with Šídák's multiple comparison test (APOE) were performed. * (p<0.05) indicates a significant difference between groups.


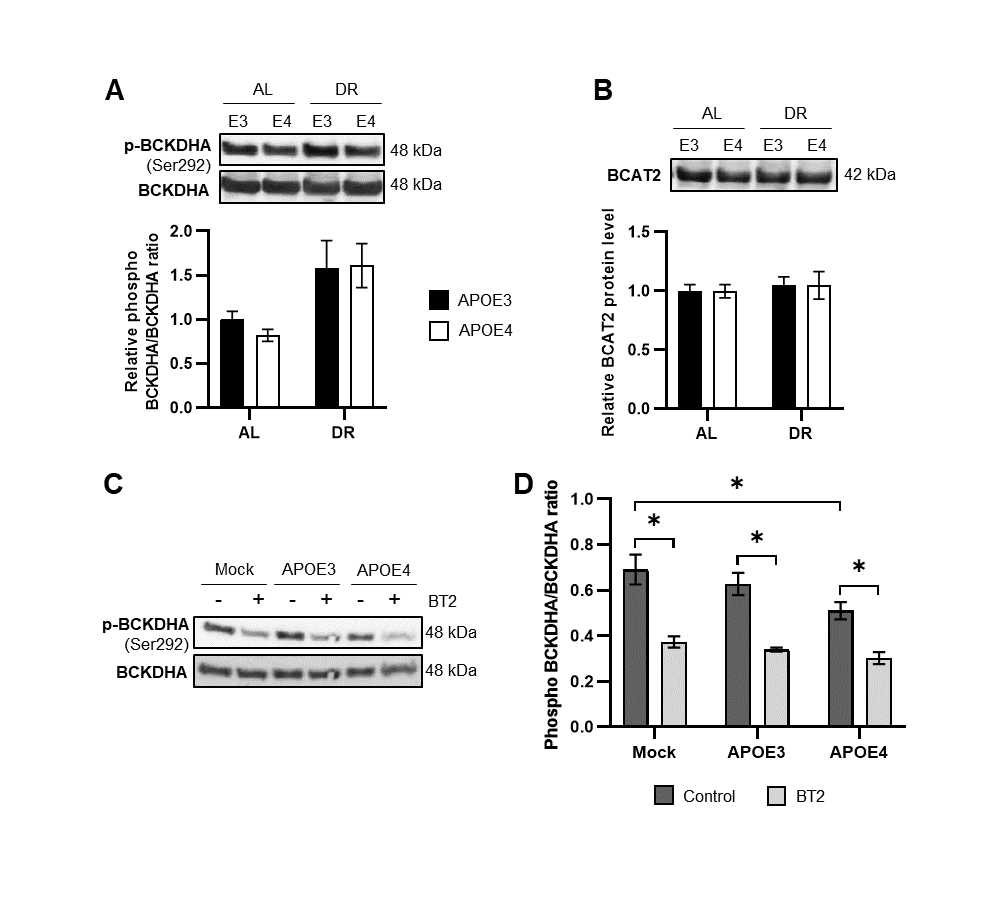


Fig. S4**: BCKDHA phosphorylation and BCAT2 protein levels in muscle tissue from male APOE TR mice and BCKDHA phosphorylation in** HepG2 cells treated with the BCKD kinase inhibitor BT2.

(**A**) In muscle, the ratio of phosphorylated to total BCKDHA was slightly increased by DR (p=0.040; one-way ANOVA) but did not differ between the groups (Šídák's multiple comparison test). (**B**) No differences were observed in the BCAT2 protein levels between the groups (p=0.948, one-way ANOVA). The data are shown relative to the APOE3 AL group (means ± SEMs, n=5-6). (**C**) Representative Western blot images and (**D**) densitometric analysis showing reduced BCKDHA phosphorylation after BT2 treatment in APOE-transfected HepG2 cells (mock: p<0.001; APOE3: p=0.001; APOE4: p=0.015). The untreated control group of APOE4 cells showed a lower phosphorylation ratio than the untreated mock control group (p=0.044; one-way ANOVA, Šídák's multiple comparison test). The data are shown as the means ± SEMs (n=4). * (p<0.05) indicates a significant difference.
